# Progress in Surgery

**Published:** 1900-04-14

**Authors:** 


### Progress in Surgery.

### GENITO-URINARY ORGANS.

(*Continued from page 435, Vol. XXVII.*)

**Pyelonephrosis.**—Carlier[1] performed nephrotomy upon a man who had suffered from several attacks of gonorrhœa, and had pyuria. The enlarged left kidney contained a pint of pus. The patient had a stricture also, and internal urethrotomy was practised. A renal fistula remained, and a year and a half later Carlier found an hour-glass-shaped abscess occupying the left iliac fossa and the left side of the scrotum. The pus probably had developed in the fatty capsule of the kidney, and then in the iliac fossa and inguinal canal. The abscess was opened, and nephrectomy performed after the other kidney had been found to be secreting healthily by the methylene blue test. The kidney was small and the capsule so dense that the kindney had to be shelled out of it. The kidney contained three purulent cavities. Recovery followed. Leonard Freeman[2] records some instructive cases of this affection. In one a man of about thirty-five had suffered for several years from prostatic abscesses, following a perineal operation for stone. After the relief of the prostatic condition great irritability of the bladder persisted, with moderate fever, debility, and loss of weight. The muscles were rigid over the right kidney, but no tumour could be felt. Exploratory nephrotomy revealed the presence of five separate cavities in the kidney, containing several pints of pus. Drainage was employed, and the bladder symptoms at once disappeared. Freeman, in connection with the case, calls attention to the fact, not widely enough appreciated, that irritability of the bladder may be due to kidney affections. In opening collections of pus about the kidney, it must be ascertained whether there are more cavities than one. Two cases are recorded of suppurative nephritis following parturition. In each case the urine was cloudy with pus in suspension, and there were pain and tenderness in the right loin. A free incision in each case revealed an enlarged softened kidney with numerous foci of infection scattered throughout its substance. Recovery followed drainage. These cases indicate that even cases of suppurative nephritis with disseminated foci of pus may be cured by drainage. The same writer mentions one case of pyelitis, and another of suppurative nephritis, due to the colon bacillus, both following fulminating appendicitis. Two other cases of pyelonephritis, due to the colon bacillus, followed the catheterisation of paretic bladders. They ended fatally. Urotropine and salol are recommended for these colon-bacillus infections.

**Movable Kidney.**—Three other cases of Freeman's are worth allusion to under this heading. In one a movable kidney in a well-nourished young woman of twenty-five was associated with the presence of an immense number of hyaline, granular, and epithelial casts, without albumin. After nephrorrhaphy the casts rapidly disappeared, and it seems likely that the operation averted serious danger of progressive renal disease. In another case, that of a woman of thirty-five, a right movable kidney was associated with severe sciatica which had resisted all treatment. This immediately disappeared after nephrorrhaphy. The third case was that of a woman of the same age, who had had severe dragging pains in the pelvis, back, and thighs, and bladder irritability, following soon after a fall fourteen years before. A gynæcologist had removed the uterus, tubes, and ovaries without relieving her. The annoying pains were abolished by fixing both kidneys, which were movable. The moral of such a case is obvious. Büdinger[3] has written recently on the ætiology of floating kidney. He rejects the idea that the location and mobility of the kidney cannot be determined by researches upon the cadaver, based on the hypothesis that the intra-abdominal pressure which produces these changes is absent. Authorities differ much as to the normal amount of mobility of the kidney. From a careful study of sixty cadavera Büdinger found that the kidneys were fixed in eleven cases, and in the remaining were more or less movable. The proper suspensory ligament and principal means of support of the kidney he considers to be the plane of connective tissue which extends from the kidney around the posterior aspect of the renal vessels, fusing in part with the connective tissue surrounding the aorta, and in part with the fascia covering the lumbar portion of the diaphragm. Relaxation of this ligament, and of the connective tissues surrounding the kidney, results in floating kidney, and is caused generally by injury. The neighbouring organs act as auxiliaries in increasing the mobility. In treating the condition the importance of obliterating the sac-like space in which the floating kidney lies is emphasised. Bruce Clarke[4] draws attention to the long period of time which may elapse between the first symptoms of movable kidney and the symptoms which indicate that serious and possibly irreparable damage has been done to the affected organ. He quotes an illustrative case in which he removed a kidney, which was little more than a bag of pus, from a woman of thirty-two, who had suffered off and on for sixteen years. The pain in the lumbar region in the earlier years had been thought to be due to weakness of the back. She married at twenty-seven and had had two children since. During each pregnancy pus appeared in the urine, and she had several attacks of pain. After the second the pain continued, and the kidney was felt to be enlarged. She was cured by nephrectomy. Clarke remarks that there can be no reasonable doubt that if nephropexy had been performed years before, the integrity of the kidney would have been preserved, and much suffering avoided. The same writer has collected notes of twenty cases in which pregnancy has been the starting-point of suppuration in movable kidney, and is assured that no woman with symptoms of movable kidney ought to get married until it has been fixed in its place. Again, he states that movable kidneys contain uric acid stones far more frequently than do normal kidneys. That the mobility of the kidney has been the predisposing cause of the formation of the stones in many of these cases, Clarke thinks is clear. It can hardly be supposed that a stone firmly impacted in the cortex or calyces will cause mobility of the kidney, and the absence of stone in the other kidney is in favour of the mobility of the affected organ having a causative effect. It is pointed out th[illegible] the colloidal cementing substance required for the [illegible]rmation of calculi may be supplied as a result of [illegible]mmation of the movable organ producing mucu[illegible] pus. Clarke remarks also that it is in the earliest stage of the disease, when the kidney has just begun to shift its position, and is liable to entangle its vessels with surrounding structures, that it is so prone to attacks of congestion. Then is the time it should be fixed in place. When its vessels have been lengthened by long pulling, and it can swing backwards and forwards on its moorings easily, it rarely comes to much harm, and is oftentimes then not a source of inconvenience, and can be left alone.

**Rupture of the Kidney.**—Bland Sutton[5] has reported to the Clinical Society a successful case of nephrectomy for this injury. The patient was a man of thirty-five, who had been run over by a cab. There were no superficial traces of injury, but marked signs of internal bleeding, and a large, tense, and ill-defined swelling in the right loin. Three hours after admission to the hospital the signs of bleeding, and the collapse, became so marked, and so much blood appeared in the urine, that there could be no doubt that the kidney had been severely injured. An incision was made in the right linea semilunaris, and the kidney was found to be completely torn across at the junction of the lower and middle thirds. The fragments were removed with the surrounding clots, and the cavity left lightly stuffed with gauze. The patient was discharged in four weeks. Sutton remarked that home literature contained very few records of primary nephrectomy for rupture of the kidney. A study of the *Centralblatt für Chirurgie* showed that far too many cases were recorded from post-mortem examinations. It was much to be regretted that the operation should not be done immediately after the injury, so as to check the hæmorrhage at once. We may refer here to Morris's views on this subject, as enumerated in the *Clinical Journal*, 1894. He there writes, "The presence of a distinct and increasing swelling in the renal region makes an early incision through the loin imperative: it is the only way to cut short the progress of the case, to limit the extravasation into the peri-renal tissues, to check or prevent suppuration, to prevent sloughing of the peritoneum, and to save the life of the patient." Critchley Hinder[6] records another case of ruptured kidney successfully treated by operation, from Australia. A boy of twelve fell, striking his right flank against an iron shed. He walked home in great pain. Seen two hours later, he had a small and weak pulse, and great pain with the least movement. The right flank was tender and rigid. During the next two hours he passed ten ounces of pure blood from the bladder, and shortly after that clear urine. It was concluded that the ureter was plugged by a clot. Later the bleeding recurred. The kidney was explored the next day. The lower two-fifths were found crushed, with pieces of kidney hanging loose. These pieces were removed. The wound was packed with gauze. The gauze was removed on the third day, and on the fourth two ounces of urine escaped from the wound. Within a fortnight the wound had closed and the urine was clear. The prognosis in cases of renal injury is much worse if cystitis exists, for the inflammation easily passes up to the damaged kidney, and is aggravated by the blood which has collected in the bladder undergoing decomposition. The sound kidney may also become affected with suppurative nephritis similarly.

**Rupture of the Ureter or Renal Pelvis.**—E. Percy Paton[7] records an interesting case of this injury. A man, aged thirty-six, was admitted to hospital ten hours after a fall in which he had struck the left side of his abdomen. He walked into the surgery. There was a fracture of the left twelfth rib and slight hematuria. During the next few days there was vomiting, and displaceable flank dulness, but later he improved so much that he was allowed to get up. On the 12th and 13th day the pain returned and gradually increased, and a distinct tumour could then be made out. It was dull and elastic, and extended from the left loin to the iliac fossa. A lumbar incision was made, and from one to two pints of clear urinous fluid escaped. No opening either into the kidney, pelvis, or ureter could be found. Much urine escaped through the incision for several days. Eight weeks after the operation he was well, with normal urine, the pyuria, which had developed as the wound closed, having then disappeared. The injury must have been rupture of the ureter or renal pelvis. Paton refers to other cases like his own in which the ideal treatment of exposing the rent and suturing it could not be adopted, as there was no ground for the diagnosis until some time after the injury. The small amount of irritation to which normal urine entravasated into the tissues gives rise is commented upon. In this case the temperature never exceeded 100·5.

**Injury of the Ureters from Surgical Operations.**—Mac Monagle[8] contributes a paper on these injuries, and their treatment. He enumerates the following methods of restoring the continuity of the ureter in such cases. 1. Invagination of the proximal into the distal end. 2. Transverse end-to-end anastomosis. 3. End-to side anastomosis. 4. Implantation into the bladder. 6. Implantation into the bowel. 7. Crossed anastomosis—*i.e.*, switching the ureter into its fellow of the opposite side. These methods are available when the injury is discovered at, or immediately after, an abdominal operation, or when the vaginal operation for uretero-vaginal fistulæ has failed. Of the first of these—the invagination of the ends, Mac Monagle records three of his own cases, and alludes to one of Robson's and one Winslow's. Two of the author's cases made an excellent recovery. Seven cases of transverse end-to-end anastomosis have been reported, and four of these were completely successful, and all successful as far as the ureter was concerned. Elastic catheters have been inserted to facilitate the suturing. They were removed either through the urethra and bladder, or from the ureteral wound before inserting the last sutures. The end-to-side method has been very successful. It was conceived by Van Hook. Oblique end-to end anastomosis has also been carried out successfully. The obliquity of the ends prevents stenosis, and requires less loss of ureteral length than Van Hook's method. Implanation into the bladder has been performed intraperitoneally and extraperitoneally, the former ten times, with no deaths, the latter four times, with one death. Most of the injuries under discussion have been due to a displacement of the ureters by a tumour arising deep in the pelvis, or to carcinoma, involving the broad ligaments. They occur most often during vaginal hysterectomies. The repair of complete transverse wounds usually succeed in man. The outer fibrous sheath of the ureter carries the chief blood supply, and is closely connected with the peritoneum. Stripping off the peritoneum is therefore liable to lead to necrosis of the ureteral wall. Implantation into the bladder is the operation of choice when the injury is below the pelvic brim, and too much tension does not result. To prevent tension the bladder may be separated from the rami of the pubis and stitched higher in the pelvis. Nephrectomy should be regarded only as a last resource, though it has frequently been successful. One successful case of implantation into the bowel has been recorded. Implantation on the skin can be used to gain time if the patient's condition is critical. Tying the ureter, with a chance of atrophy of the kidney, is stigmatised as being unsurgical. It will be recognised that Mac Monagle's paper, from which the above remarks have been gleaned, is one of great interest and importance.

[1] Brit. Med. Jour., Nov. 1899. [2] New York Med. Jour., Oct., 1899. [3] Vide Annals of Surgery, Dec. 1899. [4] Clinical Jour., Jan., 1900. [5] The Lancet, Nov., 1899. [6] Australasian Med. Gazette, Nov., 1899. [7] Brit. Med. Jour., Jan., 1900. [8] Amer. Jour. Med. Sci., Dec., 1899.

